# Ten‐Year Results of a Prospective Case Series on Immediately Provisionalized One‐Piece Alumina‐Toughened Zirconia (ATZ) Oral Implants for Single‐Tooth Restoration and Three‐Unit Fixed Dental Prostheses

**DOI:** 10.1111/jcpe.70156

**Published:** 2026-06-17

**Authors:** Ralf‐Joachim Kohal, Sebastian B. M. Patzelt, Benedikt C. Spies, Taro Iwasaki, Kei Kubochi, Kirstin Vach, Marc Balmer

**Affiliations:** ^1^ Department of Prosthetic Dentistry, Faculty of Medicine, Center for Dental Medicine Medical Center – University of Freiburg Freiburg Germany; ^2^ Private Dental Clinic Zimmern ob Rottweil Germany; ^3^ Department of Fixed Prosthodontics Nihon University School of Dentistry Chiyoda‐Ku Tokyo Japan; ^4^ Institute of Medical Biometry and Statistics, Medical Center – University of Freiburg Freiburg Germany; ^5^ Clinic of Reconstructive Dentistry, Center for Dental Medicine University of Zurich Zurich Switzerland

**Keywords:** clinical investigation, dental implants, fixed dental prosthesis, prospective, single crown, zirconia implant

## Abstract

**Aims:**

The intention of this 10‐year prospective case series investigation was to clinically and radiographically investigate the outcomes of a one‐piece alumina‐toughened zirconia oral implant.

**Materials and Methods:**

Twenty seven patients received one implant each for single crown restorations (SC), while 13 patients received two implants for a three‐unit fixed dental prosthesis (FDP). Eighteen implants were placed in the upper jaw, with four implants in the upper anterior region and nine in the premolar region. The 35 implants of the lower jaw were placed in the premolar (*n* = 15) or molar region. Follow‐up assessments were performed at final restoration insertion (RI), after 1, 5 and 10 years. Peri‐implant soft tissue parameters were evaluated, and the peri‐implant bone assessed at implant placement, final RI and at the 1‐, 5‐ and 10‐year follow‐ups. Statistical analysis was conducted to compare differences over time and between groups (*p* < 0.05).

**Results:**

Three implants were lost in the SC patient group and none in the FDP group, yielding a cumulative survival rate of 94.3% for SC and FDP combined. The mean marginal bone loss of the remaining implants was 0.99 mm (implant placement to 10 years: *p* < 0.001) (0.61 mm for SC and 1.26 mm for FDP; difference: *p* < 0.05). Probing depth at implant sites increased (*p* = 0.006), the plaque index remained stable (*p* = 0.639) and the bleeding showed a significant decrease (*p* < 0.001), each from RI to the 10‐year follow‐up after loading. Patients experienced significant improvements in function, aesthetics, sense, speech and self‐esteem from pretreatment up to the 10‐year follow‐up (all *p* < 0.05).

**Conclusions:**

The high implant survival rate observed after 10 years substantiates the clinical applicability of the investigated implant system.

## 1 Introduction

Over the past decade, zirconia implants have attracted attention as a metal‐free alternative to titanium, the gold standard in oral implantology (Mohseni et al. 2023; Santmartí‐Oliver et al. 2023). Preclinical studies demonstrated reliable osseointegration and functional loading capacity of zirconia implants (Bethke et al. 2020; Pieralli et al. 2018). Patients may prefer zirconia implants due to their tooth‐like colour and aesthetic advantages (Zuercher et al. 2024). In addition, the use of zirconia implants avoids the potential risk of titanium hypersensitivity (Jacobi‐Gresser et al. 2013; Sicilia et al. 2008). Zirconia may reduce plaque accumulation, potentially lowering the risk of peri‐implant infection (Hosoki et al. 2016; Scarano et al. 2004).

Clinically, zirconia implants are used for single crowns (SCs) and short‐span fixed dental prostheses (FDP), showing short‐term survival rates comparable to titanium implants (Gul et al. 2024). Mid‐term studies with follow‐up periods of up to 5 years report favourable outcomes (Roehling et al. 2023), whereas long‐term data of ≥ 10 years remain limited (Borgonovo et al. 2021; Karapataki et al. 2023; Kohal, Komine, et al. 2025; Kohal, Lith, et al. 2025; Roehling et al. 2026; Steyer et al. 2021). Mechanical performance of zirconia relies on 3Y‐TZP properties for fracture resistance (Piconi and Maccauro 1999), though low‐temperature degradation (LTD) raises long‐term stability concerns (Chevalier and Gremillard 2009). Alumina‐toughened zirconia (ATZ) improves resistance to LTD while maintaining osseointegration and fracture resistance (Gremillard et al. 2018).

Accordingly, this clinical case series aimed to evaluate the clinical, radiographic and patient‐reported outcomes of a one‐piece ATZ ceramic implant system used for SC and short‐span FDPs 10 years after loading with the survival rate as primary endpoint. The implants in this series were placed in the anterior and posterior areas of the upper and lower jaw.

## 2 Materials and Methods

### 2.1 Study Design, Participants, Investigated Implant System and Procedures

The study was conducted at the Department of Prosthetic Dentistry, University Medical Center Freiburg, Freiburg, Germany. This single‐centre case series received ethical approval from the Medical Center—University of Freiburg, Germany (ref. 354/07, 354/1386‐S1‐retro), and all participants provided informed consent. Forty consecutively recruited patients (recruitment period: January 2008–July 2011; 20 females, aged 18–70) requiring SC or 3‐unit FDP restorations were included.

The study adhered to the STROBE guidelines relevant for descriptive observational studies (accessed 04 December 2025) and the Declaration of Helsinki (World Medical Association 2025).

Further detailed information on inclusion criteria, implant system, surgical and prosthetic procedures can be found in Appendix 2.1 of Supporting Information.

### 2.2 Follow‐Ups and Outcomes Measures

#### 2.2.1 Clinical Parameters, Peri‐Implant Health and Disease Classification, Survival and Success Criteria

Follow‐ups were performed at definitive restoration insertion (RI), 1, 5 and 10 years. The following clinical parameters were recorded with a periodontal probe (CP‐12 Colour‐Coded Probe, HuFriedy, Frankfurt, Germany) at implant and adjacent teeth: modified Bleeding Index (mBI), modified Plaque Index (mPI) (Mombelli et al. 1987), bleeding on probing (BOP), Probing depth (PD) and Clinical attachment level (CAL; reference point: crown margin). All measurements were taken at 4 sites (mesiobuccal, midbuccal, distobuccal, midlingual) and recorded to the nearest millimetre. For the analysis, the mean values were used.

According to the classification proposed by the World Workshop (Berglundh et al. 2018), peri‐implant health and disease were evaluated. With the absence of erythema, BOP, swelling and suppuration, peri‐implant health was recorded (Araujo and Lindhe 2018). When bleeding on gentle probing, with additional signs such as erythema, swelling or suppuration was identified, peri‐implant mucositis was diagnosed (Heitz‐Mayfield and Salvi 2018). Peri‐implantitis was diagnosed based on clinical indicators of inflammation, including bleeding on probing, suppuration, increased PDs, mucosal recession and radiographic evidence of bone loss relative to prior assessments (Schwarz et al. 2018).

The mere remaining of implants in situ was defined as implant survival. Implant success was defined according to contemporary consensus criteria, combining implant survival, absence of recurrent peri‐implant infection accompanied with suppuration, stable marginal bone levels beyond initial remodelling, absence of clinical symptoms (no mobility of the implant, no signs of pain, no foreign body–like sensation, no sensory disturbance) (Albrektsson et al. 1986; Buser et al. 1990; Karoussis et al. 2003). In contrast, implant failure was defined as the removal or loss of an implant following its placement.

Technical complications of the restorations were not included in the definition of implant success.

#### 2.2.2 Radiographic Assessment

Radiographs were taken using the long‐cone parallel technique with customized x‐ray filmholders. Image calibration was performed based on a known reference dimension using the open‐source software ImageJ (National Institutes of Health, Bethesda, MD, USA). The distance from the first bone‐to‐implant contact to a defined reference point was measured. Changes in marginal bone levels at implants and adjacent teeth were calculated at each follow‐up. For bone level analysis, the mean of mesial and distal measurements was used.

#### 2.2.3 Technical Parameters

The clinical quality of implant‐supported restorations (ISRs) was assessed using an adapted version of the US Public Health Service (USPHS) (Cvar and Ryge 2005) evaluation system. For detailed information on technical assessment see Appendix 2.2.3 of Supporting Information.

#### 2.2.4 Patient‐Reported Outcome Measures

Patient‐reported outcomes (function, aesthetics, sensation, speech and self‐esteem) were evaluated using self‐administered visual analogue scales (VASs) (Spies et al. 2015). For standardization, patients indicated their subjective perceptions by marking a 10‐cm line without numeric labels, where the left end represented ‘poor satisfaction’ (0%) and the right end ‘excellent satisfaction’ (100%). The marks were measured with a ruler, with 1 mm corresponding to 1%.

#### 2.2.5 Statistical Analyses

Linear mixed models with random intercepts analysed soft/hard tissue outcomes and patient‐reported outcome measures (PROMs), evaluating effects of time, site and restoration type. Pairwise comparisons were adjusted using Scheffé's method; significance was set at *p* < 0.05. To account for potential ceiling effects, separate variances were assumed for each time point. Since measurements involved three positions per patient (implant and two neighbouring teeth), patients were treated as clusters, with clustering applied individually for each variable. Pairwise comparisons were adjusted for multiple testing using Scheffé's method. The level of significance was set to *p* < 0.05. Statistical analyses were performed with STATA (19, StataCorp LP, College Station, TX, USA) via the *xtmixed* command.

## 3 Results

Data from the 5‐year follow‐up have been reported previously (Kohal et al. 2020) and will not be presented here.

### 3.1 Patient Recruitment, Demographics and Characteristics; Implant Distribution

A total of 284 patients applied for study participation. Of those, 89 were assessed for eligibility. Forty‐eight patients had to be excluded, resulting in 41 enrolled patients. Patients were allocated to two groups: SC group (*n* = 28) and FDP group (*n* = 13 patients; 26 implants). In the SC group, one patient withdrew before treatment, leaving 27 patients for implant placement. Three implants were lost prior to reconstruction, resulting in 24 patients at prosthesis insertion and 1‐year follow‐up. After dropouts, 22 patients remained at 5 years and 19 at the 10‐year follow‐up.

**FIGURE 1:**
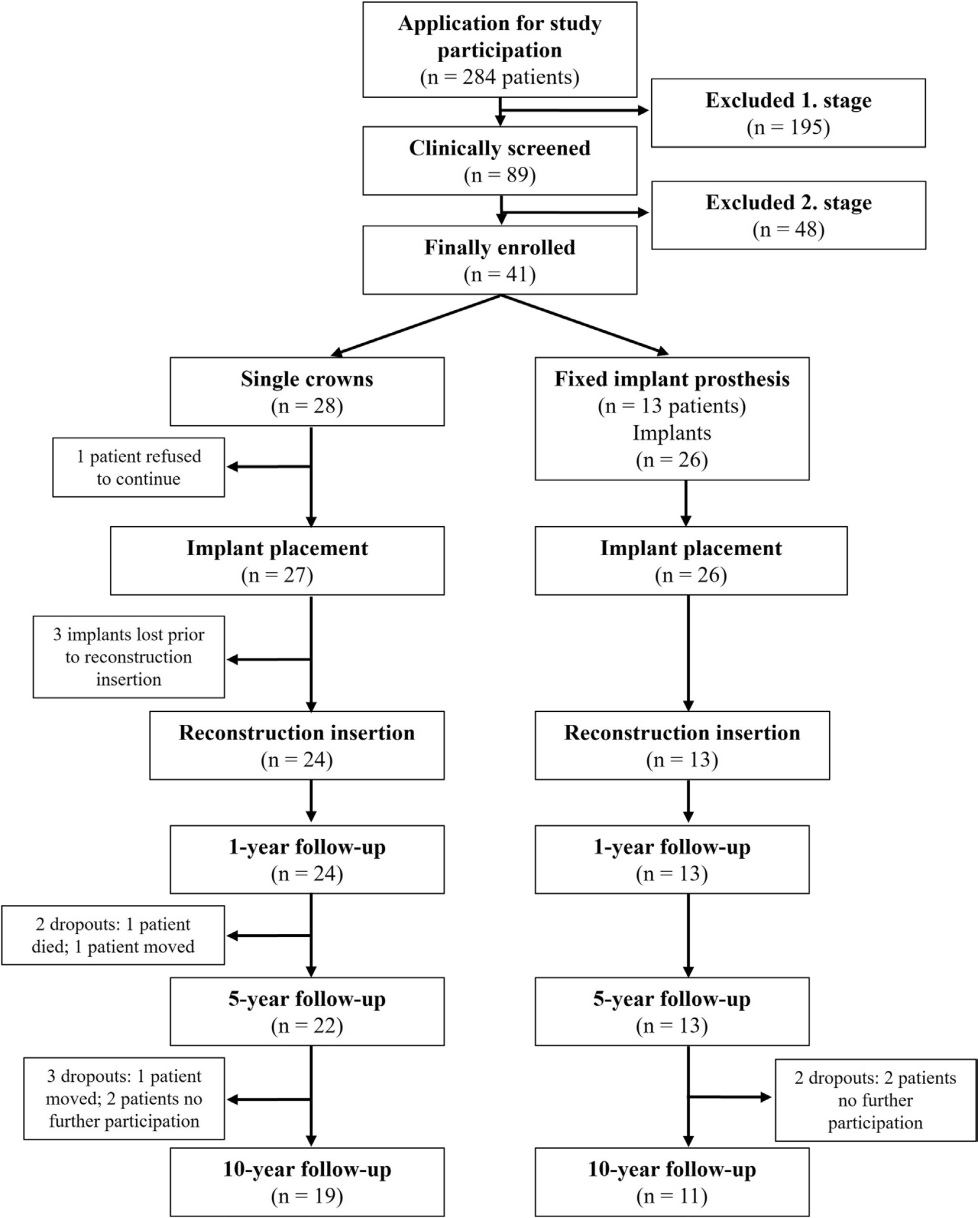
Flow chart of patient recruitment, allocation, treatment and follow‐up.

In the FDP, all 13 patients received implant placement and reconstruction, and all were available at 1‐ and 5‐year follow‐ups. Two patients were lost to follow‐up, resulting in 11 patients at 10 years. Figure 1 presents a flow chart with this information.

The patient characteristics and reasons for tooth extraction can be found in Appendix 3.1 of Supporting Information and Table S1.

**FIGURE 2:**
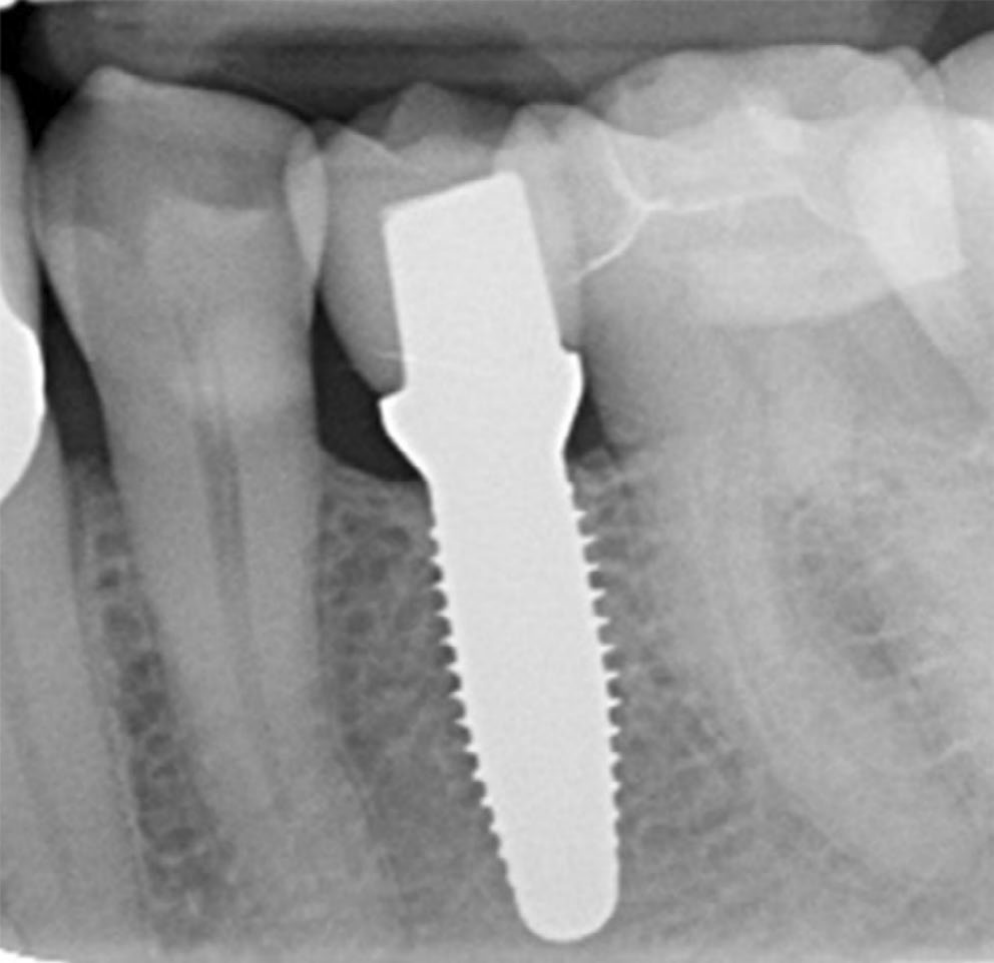
Corresponding radiograph of the peri‐implant bone situation of the SC implant at the 10‐year follow‐up.

**FIGURE 3:**
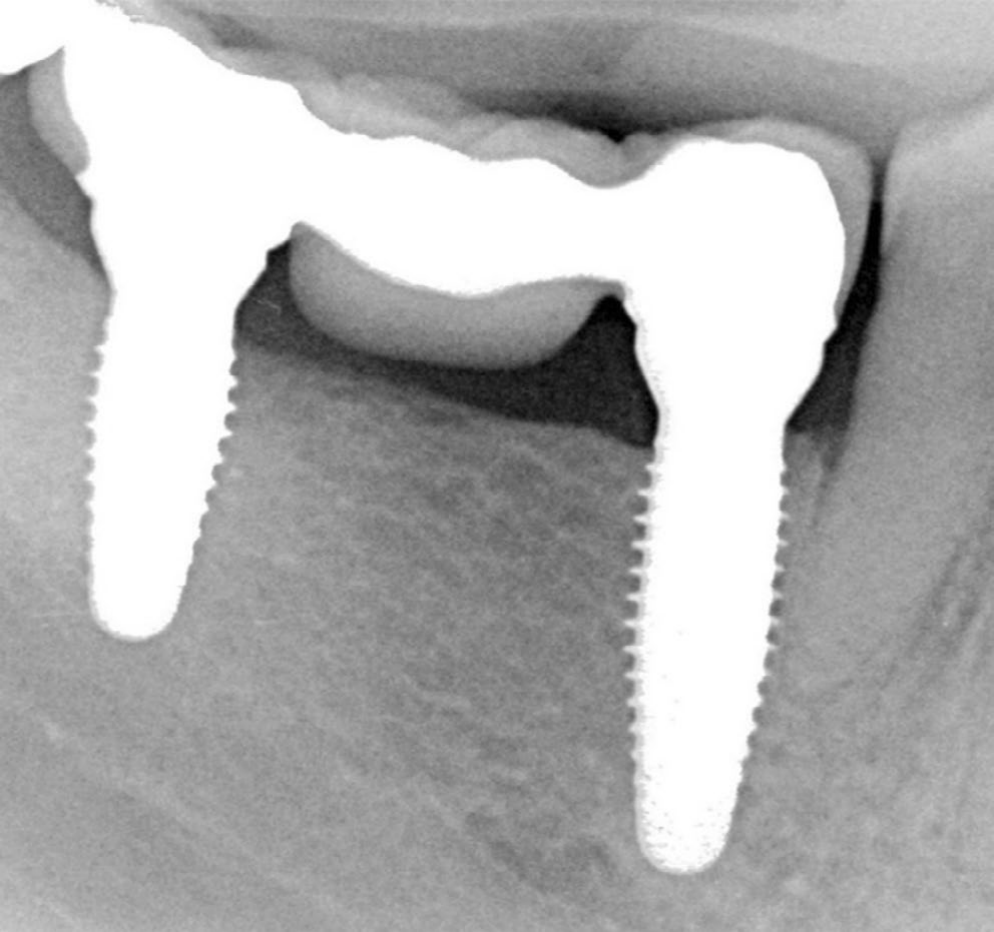
Corresponding radiograph of the peri‐implant bone situation of the two implants supporting an FDP at the 10‐year follow‐up.

Forty patients (20 women, 20 men) received a total of 53 zirconia implants: 27 SC (Figure 2 and Figure S1) and 26 supporting 3‐unit FDPs (Figure 3 and Figure S2). Two implants were inserted immediately post‐extraction; the rest in healed sites. All implants achieved primary stability ≥ 30 Ncm and were immediately temporized. The implant distribution is presented in Table S2.

### 3.2 Status of Follow‐Up and Primary Outcome: Implant Survival (Table 1)

**TABLE 1:** Status of follow‐up.

Time	No. of patients	No. of implants	No. of implant losses	Survival (%)	Survival worst case scenario (%)
Baseline to restoration insertion (RI)	40	53	3	94.3	94.3
At RI	37	50	0	94.3	94.3
RI to 1‐year follow‐up	37	50	0	94.3	94.3
1‐ to 5‐year follow‐up	35	48	0 (2^a^)	94.3	90.6^a^
5‐ to 10‐year follow‐up	30	41	0 (9^a^)	94.3	77.4^a^

^a^ Survival when the implants of the dropped‐out patients are counted as losses.

Three implants in the group of SC did not integrate and were removed before RI. These implants were located in the lower molar and premolar regions (see Table S2) in two females and one male. Hence, 50 implants were restored. Thirty‐seven out of 40 patients were seen at the 1‐year follow‐up, 35 at the 5‐year follow‐up (22 SC, 13 FDP) and 30 patients could be evaluated at the 10‐year follow‐up (19 SC, 11 FDP; Table 1). Between the 1‐year and 5‐year follow‐up, one patient died and one patient moved to an unknown location (both with one implant each). After the 1‐year follow‐up, no further implant loss occurred, which led to a cumulative implant survival rate of 94.3% after 5 years. Between years 5 and 10, five more patients (3 SC, 2 FDP) dropped out due to moving away (1) or because they had no interest in further participation (4).

At the 10‐year follow‐up, no patient reported on clinical symptoms such as implant mobility, pain at implant sites, foreign body‐like sensation or sensory disturbance.

### 3.3 Clinical Parameters

The changes in peri‐implant soft tissue parameters over time are shown in Table S3. At implant sites, the mean PD increased over the period from RI to the 10‐year follow‐up (*p* = 0.006). The mean PD at implant sites was usually significantly higher than around tooth sites. The mean CAL at implant sites remained stable over the same time period (*p* = 0.841). The mPI also remained stable from RI to the 10‐year follow‐up (*p* = 0.639), whereas the mBI decreased significantly (*p* < 0.001).

### 3.4 Marginal Bone Level and Remodelling

Of the 41 remaining zirconia implants, 39 could be evaluated radiographically regarding peri‐implant marginal bone level. One patient with 1 implant refused the radiographic evaluation of his implant at 10 years. The radiograph of a further implant was not readable.

**FIGURE 4:**
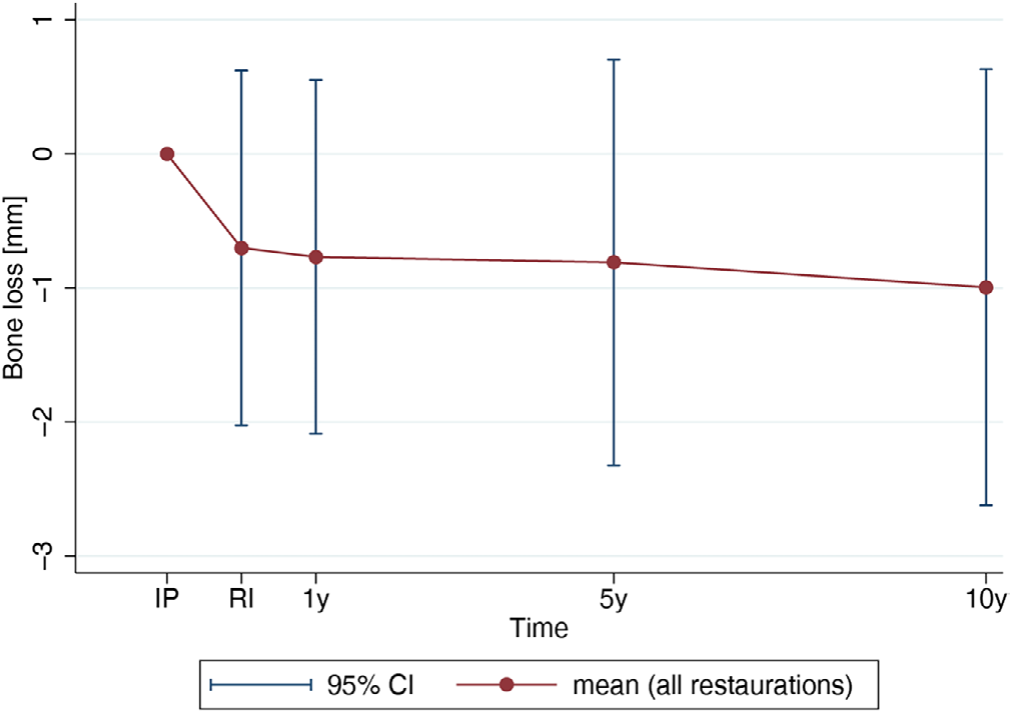
Marginal bone loss at implants irrespective of reconstruction from implant placement until the 10‐year follow‐up. 1y = 1‐year follow‐up; 5y = 5‐year follow‐up; 10y = 10‐year follow‐up; IP = implant placement; RI = restoration insertion.

The mean marginal bone loss from implant placement to RI was 0.71 mm (±0.67), 0.78 mm (±0.67) to the 1‐year and 0.99 mm (±0.83) to the 10‐year follow‐up (Table 2, Figure 4 and Figure S3). The bone loss from implant placement to the 1‐year follow‐up was statistically significant (*p* < 0.001). After the initial bone remodelling (from implant placement to the 1‐year follow‐up), mean marginal bone levels remained stable throughout the 10‐year follow‐up period.

**TABLE 2:** Marginal bone loss (in mm) at restoration insertion (RI) and at the 1‐ and 10‐year follow‐up (FUP) (mesial reference tooth, implant site, distal reference tooth).

Position	RI			1‐y FUP			10‐y FUP			Significance (*p*)		
	*n*	Mean	SD	*n*	Mean	SD	*n*	Mean	SD	IP to RI	IP to 1 year	1 year–10 years
Mesial tooth	30	0.06	0.92	35	0.11	0.63	27	0.19	0.98	0.593	0.179	0.825
All Implants	45	0.71	0.67	48	0.78	0.67	39	0.99	0.83	< 0.001*	< 0.001*	0.118*
Implants SC	23	0.39	0.71	24	0.46	0.53	23	0.61	0.70	< 0.001	< 0.001	0.409
Implants FDP	22	1.03	0.47	24	1.08	0.66	16	1.26	0.82	< 0.001	< 0.001	0.344
Distal tooth	24	0.12	0.64	25	0.35	0.58	17	0.47	0.84	0.170	< 0.001	0.593

*Note:* The marginal bone level at the day of implant placement (IP) served as baseline. Significant differences regarding the restoration type (SC or FDP) were marked with asterisk (*).

From the 1‐year to the 10‐year follow‐up, 1 implant showed bone loss of 2.9 mm (distal implant of an FDP), 4 of 1.3–1.7 mm (two SC [one SC with the loss of distal tooth which caused the bone loss] and two distal implants of FDP), 3 of 0.6 to 0.9 mm (three SC), 12 of 0.1 to 0.5 mm (six SC and six FDP) and 19 implants gained bone.

### 3.5 Peri‐Implant Tissue Conditions

At the 10‐year follow‐up, 14 SC implants fulfilled the criteria for healthy peri‐implant conditions, meaning that no bleeding on probing, inflammation or suppuration was present and the marginal bone loss did not exceed 0.5 mm (measurement error: Berglundh et al. 2018) or the reason for bone loss was the extraction of the distal tooth (1 implant). The implant of the patient who refused the x‐ray at 10 years was regarded as healthy (no bleeding, no suppuration, PD ≤ 4 mm). Two SC implants exhibited signs consistent with peri‐implant mucositis (mBI and BOP positive, one implant with increasing PD and one without increasing PD). Three SC implants suffered from peri‐implantitis (BOP and/or suppuration, all with increasing PD ≥ 5 mm). Among the 22 implants used to support FDP, 17 were classified as healthy (among those was the implant where the x‐ray could not be read: no mBI, no BOP, no suppuration, PD ≤ 4 mm). Two implants displayed peri‐implant mucositis, while three were diagnosed with peri‐implantitis.

In total, according to the success criteria mentioned above, 31 of the 41 implants were regarded as successful (success rate: 71.3%). Ten implants could only be regarded as surviving due to the signs of peri‐mucositis or peri‐implantitis.

### 3.6 Implant Restorations

The evaluated SC showed no framework or veneer fractures. All 19 crowns were regarded as successful. One FDP showed a veneer fracture up to the framework and was regarded as a failure. However, the patient objected to a renewal and it remained in function.

### 3.7 Patient‐Reported Outcome Measures

**FIGURE 5:**
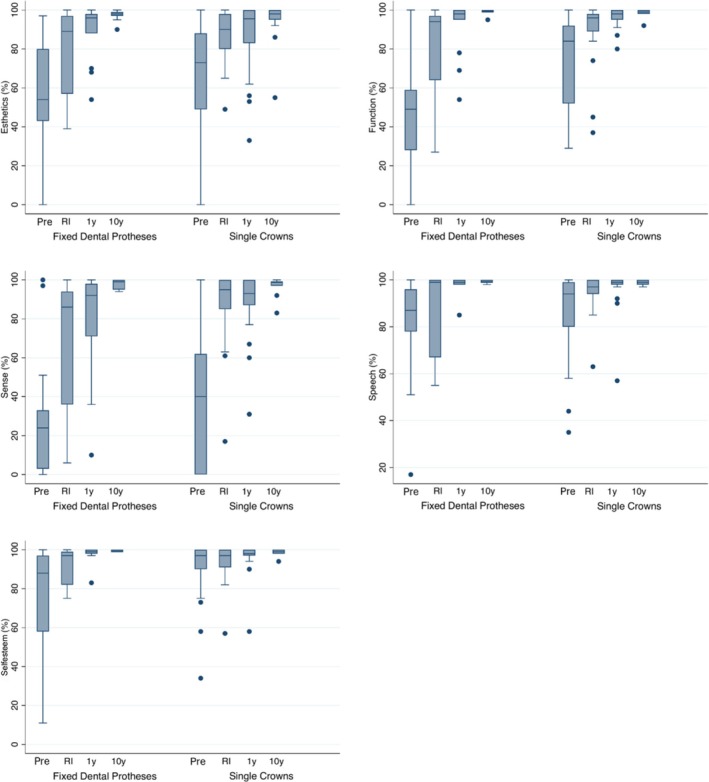
Box‐plot presentation of patient's assessment at pretreatment (PT), restoration insertion (RI) and at the 1‐ and 10‐year follow‐ups. An improvement of all parameters can be observed over the entire evaluation period.

Compared with the pretreatment situation (33.9%–85.2%), mean VAS scores showed significant improvement at the time of RI (81%–93.5%). All parameters furthermore improved significantly until the 10‐year follow‐up (Table S4 and Figure 5).

## 4 Discussion

This prospective case series provides long‐term clinical and radiographic data on one‐piece ATZ implants supporting SC and FDP. Three implants were lost prior to prosthetic restoration, whereas no further implant losses occurred after loading, resulting in a cumulative survival rate of 94.3%. This survival rate compares favourably with long‐term survival data reported for titanium implants (Kadkhodazadeh et al. 2024).

The early loss of three implants occurred during the initial phase and should be considered multifactorial. Early implant failure has been associated with a variety of patient‐, site‐ and surgeon‐related factors (Chrcanovic et al. 2016; Nyland et al. 2024). However, these factors were not evident in the present cohort (healthy patients, experienced surgeons) and therefore cannot readily explain the observed failures. In addition to surgical aspects, implant‐specific characteristics may have contributed. Zirconia implants differ from titanium implants with respect to material properties and surface modification techniques, and higher early failure rates have been reported for certain zirconia systems, particularly during earlier developmental phases (Cionca et al. 2015; Roehling et al. 2018). The one‐piece design of the investigated implant system requires precise three‐dimensional positioning and does not allow prosthetic correction of angulation, which may increase technique sensitivity. Overall, the available data do not allow identification of a single causative factor, and the observed early losses are most likely the result of a combination of surgical, biological and implant‐related factors.

One‐piece implant systems present both advantages and limitations that should be considered when interpreting these findings. A key advantage is the absence of an implant–abutment interface and the associated microgap, which has been linked to bacterial colonization and micromovement in two‐piece systems. By eliminating this interface, one‐piece implants may reduce the risk of microbial leakage and mechanical complications at the implant–abutment junction. In addition, the transmucosal configuration avoids repeated abutment disconnection and reconnection, which has been suggested to influence peri‐implant soft tissue stability and marginal bone remodelling (Koutouzis et al. 2017). However, prosthetic flexibility is reduced, as angulation corrections are not possible, and implant positioning must therefore be highly precise. In addition, restorative procedures include cement‐retained restorations. Residual cement has been identified as a potential contributing factor to peri‐implant inflammation (Düzgün et al. 2025; Staubli et al. 2017). These aspects underline the importance of careful surgical planning, meticulous prosthetic execution and appropriate maintenance protocols when using one‐piece implant systems.

Long‐term clinical investigations on zirconia implants remain limited and report conflicting results. In two 10‐year clinical investigations, Kohal, Komine, et al. (2025) and Kohal, Lith, et al. (2025) reported survival rates of 59.3% for FDP and 73.3% for SC; however, these implant systems were never introduced into the market. In contrast, Steyer et al. (2021) reported a Kaplan–Meier survival estimate of 80% after 11 years, while Borgonovo et al. (2021) after 10 years and Lorenz et al. (2019) after 7.8 years reported survival rates of 100% in smaller cohorts. A recent multicentre study described a 10‐year survival rate of 97.7% (Roehling et al. 2026). The present findings fall within this range and, to the authors' knowledge, represent the first long‐term clinical data for ATZ implants.

Marginal bone loss was approximately 1 mm after 10 years in the present investigation, which is consistent with findings reported for both titanium and zirconia implants (Mohseni et al. 2023; Moraschini et al. 2015).

Biological complications were observed in the present investigation, with peri‐implant mucositis affecting 9.8% and peri‐implantitis 14.6% of implants. While the mucositis prevalence appears lower than estimates reported for titanium implants, the peri‐implantitis prevalence falls within the range of implant‐level data reported in the literature, with global estimates of approximately 59% for peri‐implant mucositis and 18% for peri‐implantitis based on the 2017 World Workshop criteria (Reis et al. 2025). Focusing specifically on zirconia implants, the literature remains limited. Some longitudinal clinical data suggest that zirconia implants are associated with high survival rates and relatively low peri‐implantitis occurrence, with mucositis being the more common biological complication (Becker et al. 2017; Borgonovo et al. 2021; Brunello et al. 2022; Roehling et al. 2026; Steyer et al. 2021). Roehling et al. (2026) reported on a peri‐implantitis prevalence of 2.9% (1 out of 35 implants) and a peri‐implant mucositis prevalence of 5.7% (2 out of 35 implants) after 10 years. A higher peri‐implantitis prevalence with 15% (3 out of 20 implants) after 11 years was reported by Steyer et al. (2021). In the investigation of Becker et al. (2017), a total of 18 out of 52 patients presented with peri‐implantitis (34.6% on a patient level) after 2 years. After 9 years, no further peri‐implantitis cases were recorded (Brunello et al. 2022). One investigation explicitly mentioned that ‘no peri‐implantitis occurred’ in 91 two‐piece zirconia implants after a median of 5.6 years (Karapataki et al. 2023). Ruiz Henao et al. (2024) reported three cases of peri‐implantitis (18.8%) and two cases of peri‐implant mucositis (12.5%) after 5 years of follow‐up around Straumann PURE Ceramic implants. These results show that peri‐implant disease can also occur around zirconia implants similarly to titanium implants. Implant material alone does not appear to be a decisive factor, as patient‐related and clinical variables play a major role in disease development. This likely contributes to the occurrence of peri‐implantitis in the present cohort despite the presumed biological advantages of zirconia surfaces (Bienz et al. 2021; Roehling et al. 2017). While zirconia implants may support favourable soft tissue conditions, they are not free from biological complications, and appropriate risk management remains essential.

Technical outcomes were favourable, with no failures of SC and only one veneer fracture in FDP. These complication rates are lower than those reported for veneered restorations in the literature (Pjetursson et al. 2018; Sailer et al. 2018).

Limitations of this study include the absence of a randomized comparison with titanium implants and the relatively small sample size. However, a recent systematic review on zirconia implants reported sample sizes ranging from 12 to 74 patients, indicating that our patient group of 40 represents a respectable mid‐range within the current evidence base (Roehling et al. 2018). Furthermore, the prospective design, standardized follow‐up and complete documentation of outcomes strengthen the validity of the findings.

Within these limitations, the present results indicate that one‐piece ATZ implants can provide stable clinical outcomes over a 10‐year period when used for SC and short‐span FDP, with high survival rates, favourable technical performance and sustained patient satisfaction.

## Author Contributions

**Ralf‐Joachim Kohal:** conceptualization, formal analysis, funding acquisition, investigation, methodology, project administration, resources, supervision, writing – original draft. **Sebastian B. M. Patzelt:** formal analysis, investigation, writing – review and editing. **Benedikt C. Spies:** formal analysis, investigation, writing – review and editing. **Taro Iwasaki:** data curation, investigation, writing – review and editing. **Kei Kubochi:** data curation, investigation, writing – review and editing. **Kirstin Vach:** data curation, formal analysis, software, validation, visualization, writing – original draft. **Marc Balmer:** formal analysis, investigation, methodology, software, writing – original draft.

## Funding

This work was supported by Metoxit.

## Conflicts of Interest

The authors declare no conflicts of interest.

## Supporting information


**Figure S1:** Clinical situation of a SC implant (region 35) at the 10‐year follow‐up. Lateral view.

**Figure S2:** Clinical situation of implants (regions 45 and 47) supporting an FDP at the 10‐year follow‐up. Lateral view.

**Figure S3:** Marginal bone loss at implants supporting SC (red) and FDP (blue) from implant placement until the 10‐year follow‐up. IP = implant placement; RI = restoration insertion; 1y = 1‐year follow‐up; 5y = 5‐year follow‐up; 10y = 10‐year follow‐up.

**Supporting Information S1:** 2.1. Study design, participants, investigated implant system and procedures.

**Supporting Information S2:** 2.2.3. Technical parameters.

**Supporting Information S3:** 3.1. Patient recruitment, demographics and characteristics; implant distribution.

**Table S1:** (a) Patient characteristics. (b) Reasons for tooth extraction.

**Table S2:** Implant distribution for the upper and lower jaw (Tooth no. = international tooth numbering according to the World Dental Federation (FDI); Implants *n* = number of placed implants; number in brackets () = number of implant losses).

**Table S3:** Clinical parameters at restoration insertion (RI), at the 1‐ and 10‐year follow‐up. Significant differences regarding the type of restoration (SC or FDP) were marked with *. *n* = number of units, SD = standard deviation, PD = probing depth, CAL = clinical attachment level, mPI = modified Plaque Index, mBI = modified Bleeding Index.

**Table S4:** Patient's assessment of function, aesthetics, sense, speech and self‐esteem using visual analogue scales (VAS in %) at pretreatment (Pre), restoration insertion (RI) and at the 1‐ and the 10‐year follow‐ups. Significances were calculated for changes between Pre and the 10‐year follow‐up.

## Data Availability

The datasets generated and analysed during the current study are available from the corresponding author on reasonable request.
