# Supplementary material for: Ten‐Year Results of a Prospective Case Series on Immediately Provisionalized One‐Piece Alumina‐Toughened Zirconia (ATZ) Oral Implants for Single‐Tooth Restoration and Three‐Unit Fixed Dental Prostheses
Source: J Clin Periodontol. 2026 Jun 17;53(9):1403–12. doi: 10.1111/jcpe.70156 (PMC13477848; doi:10.1111/jcpe.70156)
Supplement: Supplementary file 1 [file JCPE-53-1403-s001.docx]

2.2.3 Technical parameters

Each restoration was systematically examined for the presence of ceramic fractures, marginal discrepancies, contour irregularities, esthetic deficiencies, and color mismatches. For each parameter, outcomes were categorized into four grades: Alpha, indicating the absence of any defect; Bravo, reflecting minor imperfections without clinical relevance; Charlie, denoting clinically relevant complications; and Delta, assigned when the restoration required removal due to failure. Restorations classified as Alpha or Bravo and meeting patient expectations were regarded as successful. Minor defects corresponding to a Bravo rating, such as limited chipping, were managed by intraoral polishing and did not compromise success. The presence of any Charlie rating resulted in classification as surviving but not successful. Any occurrence of a Delta rating led to classification of the implant-supported crown as a failure. During the 10-year follow-up, also retention losses were recorded.
