# Supplementary material for: Ten‐Year Results of a Prospective Case Series on Immediately Provisionalized One‐Piece Alumina‐Toughened Zirconia (ATZ) Oral Implants for Single‐Tooth Restoration and Three‐Unit Fixed Dental Prostheses
Source: J Clin Periodontol. 2026 Jun 17;53(9):1403–12. doi: 10.1111/jcpe.70156 (PMC13477848; doi:10.1111/jcpe.70156)
Supplement: Supplementary file 2 [file JCPE-53-1403-s002.docx]

2.1 **Study design, participants, investigated implant system, and procedures**

Inclusion criteria were age ≥18, sufficient bone volume to receive at least implants with a size of 3 x 9 mm, systemic health, and stable occlusion. Exclusion criteria included pregnancy, systemic contraindications (e.g., uncontrolled diabetes), the necessity of extensive bone augmentation, or compromised sites (e.g., mucosal disease, local radiotherapy). Minor augmentations, hence, were allowed.

Implant placement was performed between February 2008 and October 2011. Implants were placed in the tooth positions 17 to 48 according to the World Dental Federation (FDI). The investigated implant was a one-piece ATZ zirconia implant (Ziraldent® FR1, Metoxit AG, Switzerland) with a tapered cylindrical body, threaded apex, concave neck, and 6 mm-high triangular abutment. The alumina-toughened zirconia (ATZ) consisted of 20% Al₂O₃, 76% ZrO₂, and 4% Y₂O₃. The endosseous surface had macroscopic roughness and microscopic porosity (Ra = 1.8 µm), neck corundum-blasted (Ra = 0.2 µm), and transmucosal portion machined smooth (Ra = 0.03 µm). Available lengths were 9, 12, 14 mm and diameters were 3, 4, 5 mm. All implants were CE-certified. Pre-surgical assessment included medical history, clinical and radiographic evaluation (CBCT), and implant site analysis. Implants were inserted in healed ridges or fresh extraction sockets. Bone fenestrations were treated with guided bone regeneration [resorbable membrane (Bio-Gide^®^) and bovine bone substitute (Bio-Oss^®^, both Geistlich Pharma AG, Wolhusen, Switzerland)]. The implant placement torque was ≥ 30 Ncm.

All implants received immediate provisionalization with an eggshell temporary. These were relined with self-curing acrylic (Tab 2000; Kerr, Rastatt, Germany) and fixed using temporary cement (Freegenol; GC, Tokyo, Japan). Definitive restorations included all-ceramic SC (IPS e.max CAD LT, Ivoclar-Vivadent, Schaan, Liechtenstein) and 3-unit FDP (IPS e.max ZirCAD with IPS e.max ZirPress LT, Ivoclar-Vivadent) and these final restorations were cemented with a dual curing resin cement (Multilink Automix; Ivoclar Vivadent). After cementation, excess cement was removed using hand instruments (dental probe, scaler), interdental brushes and floss. After light-curing of 2 sec further cleaning was performed with hand instruments and then the resin was left to final set. Finally, the margins were polished using polishing burs.
