# Supplementary material for: Ten‐Year Results of a Prospective Case Series on Immediately Provisionalized One‐Piece Alumina‐Toughened Zirconia (ATZ) Oral Implants for Single‐Tooth Restoration and Three‐Unit Fixed Dental Prostheses
Source: J Clin Periodontol. 2026 Jun 17;53(9):1403–12. doi: 10.1111/jcpe.70156 (PMC13477848; doi:10.1111/jcpe.70156)
Supplement: Supplementary file 3 [file JCPE-53-1403-s003.docx]

**Supplementary Appendices Table 1.** (a) Patient characteristics. (b) Reasons for tooth extraction.

| (a) |  |
| --- | --- |
| Patient age | 47.95 ± 19.95 years (median: 46.5 years) |
| Female/male | n = 20/20 |
| ASA physical status classes | I (normal healthy) = 32 |
|  | II (mild systemic disease) = 8 |
| Smoking | none = 37 |
|  | ≤ 5 cigarettes per day = 3 |
| (b) |  |
| Reason for tooth extraction | Caries = 31 |
|  | Periodontal disease = 5 |
|  | Endodontic lesions = 4 |
