# Supplementary material for: Ten‐Year Results of a Prospective Case Series on Immediately Provisionalized One‐Piece Alumina‐Toughened Zirconia (ATZ) Oral Implants for Single‐Tooth Restoration and Three‐Unit Fixed Dental Prostheses
Source: J Clin Periodontol. 2026 Jun 17;53(9):1403–12. doi: 10.1111/jcpe.70156 (PMC13477848; doi:10.1111/jcpe.70156)
Supplement: Supplementary file 5 [file JCPE-53-1403-s005.docx]

**Supplementary Appendices Table 4.** Patient’s assessment of function, esthetics, sense, speech and self-esteem using visual analogue scales (VAS in %) at pretreatment (Pre), restoration insertion (RI) and at the 1- and-the 10-year follow-ups. Significances were calculated for changes between Pre and the 10-year follow-up.

| Assessment parameter | Pre | | RI | 1-year follow-up | 10-year follow-up | Significance (p) |
| --- | --- | --- | --- | --- | --- | --- |
|  |  |  |  |  |  | Pre →10 years |
| Function | n | 40 | 34 | 37 | 30 |  |
|  | Mean (%) | 65.3 | 86.0 | 94.7 | 98.8 | <0.0001 |
|  | SD | 27.8 | 19.7 | 9.8 | 1.6 |  |
| Esthetics | n | 40 | 34 | 37 | 30 |  |
|  | Mean (%) | 61.5 | 84.8 | 87.7 | 96.0 | <0.0001 |
|  | SD | 29.5 | 16.5 | 17.1 | 8.4 |  |
| Sense | n | 40 | 34 | 37 | 30 |  |
|  | Mean (%) | 33.9 | 81.0 | 83.8 | 97.5 | 0.0002 |
|  | SD | 33.8 | 26.7 | 23.5 | 3.4 |  |
| Speech | n | 40 | 34 | 37 | 30 |  |
|  | Mean (%) | 85.1 | 92.7 | 96.9 | 99.1 | 0.008 |
|  | SD | 19.5 | 12.3 | 7.5 | 0.8 |  |
| Self-esteem | n | 40 | 34 | 37 | 30 |  |
|  | Mean (%) | 85.2 | 93.5 | 96.8 | 99.1 | 0.0005 |
|  | SD | 23.1 | 9.2 | 7.3 | 1.2 |  |
