# Supplementary material for: Ten‐Year Results of a Prospective Case Series on Immediately Provisionalized One‐Piece Alumina‐Toughened Zirconia (ATZ) Oral Implants for Single‐Tooth Restoration and Three‐Unit Fixed Dental Prostheses
Source: J Clin Periodontol. 2026 Jun 17;53(9):1403–12. doi: 10.1111/jcpe.70156 (PMC13477848; doi:10.1111/jcpe.70156)
Supplement: Supplementary file 6 [file JCPE-53-1403-s006.docx]

**Supplementary Appendices Figure 3.** Marginal bone loss at implants supporting SC (red) and FDP (blue) from implant placement until the 10-year follow-up. IP = implant placement; RI = restoration insertion; 1y = 1-year follow-up; 5y = 5-year follow-up; 10y = 10-year follow-up
