# Supplementary material for: Ten‐Year Results of a Prospective Case Series on Immediately Provisionalized One‐Piece Alumina‐Toughened Zirconia (ATZ) Oral Implants for Single‐Tooth Restoration and Three‐Unit Fixed Dental Prostheses
Source: J Clin Periodontol. 2026 Jun 17;53(9):1403–12. doi: 10.1111/jcpe.70156 (PMC13477848; doi:10.1111/jcpe.70156)
Supplement: Supplementary file 7 [file JCPE-53-1403-s007.docx]

**Supplementary Appendices Table 3**. Clinical parameters at restoration insertion (RI), at the 1- and 10-year follow-up. Significant differences regarding the type of restoration (SC or FDP) were superscripted with *. n = number of units, SD = standard deviation, PD = probing depth, CAL = clinical attachment level, mPI = modified Plaque Index, mBI = modified Bleeding Index.

|  | **RI** | | | **1-y follow-up** | | | **10-y follow-up** | | | **Significance (p)** | | |
| --- | --- | --- | --- | --- | --- | --- | --- | --- | --- | --- | --- | --- |
|  | n | Mean | SD | n | Mean | SD | n | Mean | SD | RI 🡪 1y | RI 🡪 10y | 1y 🡪 10y |
| **PD in mm** |  |  |  |  |  |  |  |  |  |  |  |  |
|  |  |  |  |  |  |  |  |  |  |  |  |  |
| Mesial tooth | 35 | 2.19 | 0.74 | 37 | 2.28 | 0.55 | 35 | 2.18 | 0.74 | 0.427 | 0.945 | 0.395 |
| All Implants | 47 | 2.67 | 0.75 | 50 | 3.06 | 0.50 | 41 | 3.03 | 0.64 | 0.000 | 0.006* | 0.701 |
| Implants SC | 23 | 2.30 | 0.59 | 24 | 2.99 | 0.52 | 19 | 3.09 | 0.48 | 0.000 | 0.000 | 0.466 |
| Implants FDP | 24 | 3.02 | 0.73 | 26 | 3.13 | 0.48 | 22 | 2.98 | 0.76 | 0.431 | 0.958 | 0.303 |
| Distal tooth | 26 | 2.38 | 0.63 | 27 | 2.48 | 0.55 | 20 | 2.36 | 0.63 | 0.428 | 0.903 | 0.328 |
|  |  |  |  |  |  |  |  |  |  |  |  |  |
| **CAL in mm** |  |  |  |  |  |  |  |  |  |  |  |  |
|  |  |  |  |  |  |  |  |  |  |  |  |  |
| Mesial tooth | 35 | 2.68 | 1.00 | 37 | 3.05 | 1.65 | 30 | 2.66 | 0.71 | 0.301 | 0.797 | 0.198 |
| All Implants | 47 | 2.64 | 0.92 | 50 | 2.98 | 0.72 | 41 | 2.66 | 0.80 | 0.004 | 0.841 | 0.001 |
| Implants SC | 23 | 2.21 | 0.69 | 24 | 2.77 | 0.80 | 19 | 2.42 | 0.57 | 0.001 | 0.269 | 0.023 |
| Implants FDP | 24 | 3.07 | 0.92 | 26 | 3.13 | 0.58 | 22 | 2.90 | 0.64 | 0.452 | 0.510 | 0.030 |
| Distal tooth | 26 | 2.74 | 0.84 | 27 | 2.73 | 0.88 | 20 | 2.62 | 0.68 | 0.949 | 0.578 | 0.861 |
|  |  |  |  |  |  |  |  |  |  |  |  |  |
| **mPI** |  |  |  |  |  |  |  |  |  |  |  |  |
|  |  |  |  |  |  |  |  |  |  |  |  |  |
| Mesial tooth | 35 | 0.52 | 0.60 | 37 | 0.59 | 0.54 | 30 | 0.44 | 0.38 | 0.493 | 0.498 | 0.186 |
| All Implants | 47 | 0.29 | 0.41 | 50 | 0.34 | 0.46 | 41 | 0.30 | 0.31 | 0.703 | 0.639* | 0.396 |
| Implants SC | 23 | 0.08 | 0.23 | 24 | 0.22 | 0.39 | 19 | 0.18 | 0.22 | 0.114 | 0.086 | 0.766 |
| Implants FDP | 24 | 0.50 | 0.44 | 26 | 0.45 | 0.50 | 22 | 0.41 | 0.34 | 0.404 | 0.029 | 0.350 |
| Distal tooth | 26 | 0.76 | 0.61 | 27 | 0.64 | 0.53 | 20 | 0.59 | 0.39 | 0.275 | 0.048 | 0.712 |
|  |  |  |  |  |  |  |  |  |  |  |  |  |
| **mBI** |  |  |  |  |  |  |  |  |  |  |  |  |
|  |  |  |  |  |  |  |  |  |  |  |  |  |
| Mesial tooth | 35 | 0.36 | 0.39 | 37 | 0.39 | 0.44 | 30 | 0.07 | 0.15 | 0.872 | <0.001 | 0.000 |
| All Implants | 47 | 0.5 | 0.41 | 50 | 0.65 | 0.48 | 41 | 0.26 | 0.25 | 0.109 | <0.001 | 0.000 |
| Implants SC | 23 | 0.53 | 0.50 | 24 | 0.58 | 0.50 | 19 | 0.21 | 0.27 | 0.672 | 0.007 | 0.001 |
| Implants FDP | 24 | 0.49 | 0.31 | 26 | 0.70 | 0.47 | 22 | 0.30 | 0.23 | 0.051 | 0.009 | 0.000 |
| Distal tooth | 26 | 0.43 | 0.42 | 27 | 0.44 | 0.39 | 20 | 0.15 | 0.24 | 0.988 | 0.003 | 0.003 |
|  |  |  |  |  |  |  |  |  |  |  |  |  |
