# Supplementary material for: Ten‐Year Results of a Prospective Case Series on Immediately Provisionalized One‐Piece Alumina‐Toughened Zirconia (ATZ) Oral Implants for Single‐Tooth Restoration and Three‐Unit Fixed Dental Prostheses
Source: J Clin Periodontol. 2026 Jun 17;53(9):1403–12. doi: 10.1111/jcpe.70156 (PMC13477848; doi:10.1111/jcpe.70156)
Supplement: Supplementary file 8 [file JCPE-53-1403-s008.docx]

**3.1.	Patient recruitment, demographics and characteristics; implant distribution**

Five patients lost their replaced teeth due to periodontitis, 31 due to caries and 4 due to endodontic lesions. One patient reported a benign prostate hyperplasia taking a 5-alpha-reductase inhibitor. A further patient suffered from the Ehlers-Danlos-syndrome without taking any medications. Four patients took medication against hypertension. Another patient suffered from depression taking antidepressive and one patient reported trigeminal neuralgia using pain killers infrequently
