# Supplementary material for: Ten‐Year Results of a Prospective Case Series on Immediately Provisionalized One‐Piece Alumina‐Toughened Zirconia (ATZ) Oral Implants for Single‐Tooth Restoration and Three‐Unit Fixed Dental Prostheses
Source: J Clin Periodontol. 2026 Jun 17;53(9):1403–12. doi: 10.1111/jcpe.70156 (PMC13477848; doi:10.1111/jcpe.70156)
Supplement: Supplementary file 10 [file JCPE-53-1403-s010.docx]

**Supplementary Appendices Table 2.** Implant distribution for the upper and lower jaw (Tooth no. = international tooth numbering according to the World Dental Federation (FDI); Implants *n* = number of placed implants; number in brackets () = number of implant losses).

| Tooth no. | 17 | 16 | 15 | 14 | 13 | 12 | 11 | 21 | 22 | 23 | 24 | 25 | 26 | 27 | ∑ |
| --- | --- | --- | --- | --- | --- | --- | --- | --- | --- | --- | --- | --- | --- | --- | --- |
| Implants *n* | 1 | 3 | 3 | 4 |  | 1 | 2 |  | 1 |  |  | 2 | 1 |  | 18 |
| Implants *n* | 4 (1) | 2 | 4 | 3(1) |  |  |  |  |  |  | 2 | 6 | 8 (1) | 6 | 35 |
| Tooth no. | 47 | 46 | 45 | 44 | 43 | 42 | 41 | 31 | 32 | 33 | 34 | 35 | 36 | 37 |  |
